# Intravenous ketamine, propofol and propofol-ketamine combination used for pediatric dental sedation: A randomized clinical study

**DOI:** 10.12669/pjms.323.9834

**Published:** 2016

**Authors:** Dilek Gunay Canpolat, Mustafa Denizhan Yildirim, Recep Aksu, Nukhet Kutuk, Alper Alkan, Kenan Cantekin

**Affiliations:** 1 Dilek Gunay Canpolat, Anesthesiologist, Department of Maxillofacial Surgery, Erciyes University Faculty of Dentistry, Kayseri, Turkey; 2 Mustafa Denizhan Yildirim, Anesthesiologist, Department of Pediatric Dentistry, Erciyes University Faculty of Dentistry, Kayseri, Turkey; 3 Recep Aksu, Department of Anesthesiology, Erciyes University Medical Faculty, Kayseri, Turkey; 4 Nukhet Kutuk, Department of Oral Maxillofacial Surgery, Erciyes University Faculty of Dentistry, Kayseri, Turkey; 5 Alper Alkan, Department of Oral Maxillofacial Surgery, Erciyes University Faculty of Dentistry, Kayseri, Turkey; 6 Kenan Cantekin, Pediatric Dentist, Department of Pediatric Dentistry, Erciyes University Faculty of Dentistry, Kayseri, Turkey

**Keywords:** Ketamine, Propofol, Sedation, Tooth extraction

## Abstract

**Background and Objective:**

Dental treatments cannot bealways performed under local anesthesia inpediatric non-cooperative patients. For this purpose, differentanesthetic techniques have been applied to increase patient comport to dental treatments.

**Methods:**

Sixty children classified as ASA I-II, between aged 3 to 9, who were scheduled to undergo tooth extraction, were enrolled for this randomized study. Group K received 1 mg/kg ketamine, Group P received 1 mg/kg propofol, and Group KP received 0.5 mg/kg propofol plus 0.5 mg/kg ketamine intravenously for anesthesia induction.

**Results:**

Recovery time was significantly lower in Group P than Group KP. No significant differences were found between groups regarding HR, before and after the induction, at tenth minute. Fifth minute’s HR was higher in Group K than Group KP. Mean arterial pressure (MAP) values were similar at baseline, before and after the induction, and at tenth minute, whereas significantly lower values were found in Group P and Group KP than in Group K at fifth minute.

**Conclusions:**

Although ketamine, propofol and ketamine-propofol combination are effective for sedation in tooth extraction in pediatric patients, propofol may be an excellent alternative, with the shortest recovery, no nausea and vomiting, and reasonable surgical satisfaction.

## INTRODUCTION

Majority of dental treatments can be performed under local anesthesia. However, this is not always possible for pediatric non-cooperative patients with severe anxiety. In these cases, unpleasant dental experiences may lead to dental phobia when they become adults. Tooth extraction is one of the invasive procedure of dental treatment which is difficult to manage especially in children between the age of 1.5- 6 years due to fear and anxiety.^1^ Therefore, it is essential to eradicate anxiety, and to prevent a possible psychological trauma in pediatric patients. For this purpose, psychological and medical methods have been applied to increase patient compliance to dental treatment.^2^,^3^

Several sedation regimens are possible to see in literature for tooth extraction. Nitrous oxide inhalation is often used in clinics; however the success rate is low with severe anxiety.^3^ In children, intranasal midazolam-sufentanyl and ketamine-midazolam combinations or propofol and ketamine anesthesia are used intravenously for dental treatments.^1^,^4^ Ketamine and propofol alone or together have been quite excellent anesthetic regimens for the procedural sedation for the years. It has been reported that dissociative anesthesia with ketamine is quite reliable and effective, and propofol-ketamine combination leads to even better results in pediatric population.^5^ In dental procedures, midazolam, propofol and ketamine which are the short acting group of drugs were administered in children to facilitate oral rehabilitation as well.^6^

The objective of this study was to compare the anesthetic efficacy of ketamine, propofol, and propofol-ketamine combination in pediatric patients in whom dental local anesthetic attempts hadfailed due to anxiety or fear. The primary end point was recovery time, and the secondary end points included hemodynamic changes, surgeon’s satisfaction, postoperative side effects and anxiety score.

## METHODS

The study protocol was approved by the Local Ethics Committee of Erciyes University, and written consents of the parents were obtained. Sixty ASA I-II pediatric patients, between the ages of 3 to 9, who were admitted to the Erciyes University, Faculty of Dentistry, were included in this study. None of the patients were cooperative, they had severe anxiety and dental procedures could not be started despite the psychological approaches. Dental anxiety was determined according to Frankl Behaviour Rating Scale which is one of the most reliable tools developed for behavior rating of children in dental settings.^7^ The patients with serious respiratory problems, cardiac or renal failure, and the history of allergic reactions, epilepsy, and seizures were excluded from the study. Also, children undergoing lengthier restorative dental treatment were also excluded.

All the children who received EMLA (Eutectic Mixture of Local Anesthetics: Astrazeneca, London, UK) cream treatment for vascular access, unless contraindicated, and were pre-medicated using 0,1 mg/kg intravenous midazolam before taking them to operating room. Noninvasive monitoring was applied and supplemental oxygen (3-4 L/min) was administered via a nasal mask, in all cases during the procedure. Electrocardiogram (EKG), heart rate (HR), mean artery pressure (MAP), peripheral oxygen saturation (SpO_2_), capnography and respiratory rate were recorded before the procedure. End-tidal CO_2_ values were measured using a line connected to the nasal mask. Patients were randomly divided into 3 groups. Group K received one mg/kg ketamine, Group P received one mg/kg propofol, and Group KP received 0.5 mg/kg propofol plus 0.5 mg/kg ketamine intravenously for sedation.

After the induction, and every five minutes during operation, MAP, HR, respiratory rate, RSS (Ramsey Sedation Scale, 1: Nervous, agitated and/or restless, 2: cooperative, orientated, quiet patient, 3: obeying orders, 4: sleeping, responding immediately to the glabellar stimulation and high voice, 5: sleeping, responding slowly to the glabellar stimulation and high voice, 6: no response to any kind of stimulations)^8^, SPO_2_, end-tidal CO_2_ levels were recorded. Half of the initial drug dose was repeated when RSS was lower than 4, or the HR or MAP levels of patients were 20% higher than their basal values. 3-4 ml of local anesthetic solution (Ultracain D-S, Sanofi Aventis, İstanbul, Turkey) was applied for each tooth to be extracted.

Side effects such as hypoxia, respiratory depression, agitation, arrhythmia, bradycardia, hypotension or hypertension, the increase of secretions, shivering, nausea and vomiting, and hallucination were also recorded during and two hours after the procedure. Respiratory depression was defined as a respiratory rate less than 8 breats/minute or an apnea lasting longer than 15 seconds. A 20% increase in basal line values was regarded as hypertension, while a 20% decrease in basaline values was regarded as hypotension. A decrease in SPO_2_ that was less than 90% was regarded as hypoxia. A Stewared recovery score of 7 was recognized as the end of the recovery time (Consciousness, Awake: 3, Response to verbal stimuli: 2, Response to tactile stimuli: 1, Not responding: 0; Motor, Moves limbs purposefully: 2, Non-purposeful movement: 1, Not moving: 0; Airway, Cough on command or cry: 2, Maintains good airway: 1, requires airway assistance: 0).^9^

At the end of the procedure, the same anesthesiologist enquired about the surgeon’s satisfaction whereas surgeon was blinded to anesthetic technique. The satisfaction level was evaluated as follows: Good: 3 points, Mild: 2 points, Bad: 1 point.^10^ Similarly, anxiety was evaluated with a four-point scale: 1=calm and cooperative; 2=anxiosus but could be reassured: 3= anxious and could not be reassured: 4=crying or resisting.^11^

We had three groups with 10 patients in each, a total of 30 patients, as our preliminary study to find the sample size of this study. The primary end point of this study was to evaluate recovery times. We determined that α = 0.05 and β = 0.05, a sample size of 12 patients per group was required to find a difference in recovery time of 5.8 min and 3.4 standard deviation.

### Statistical Analysis

Shapiro-Wilk test was used to assess the normality of data. Comparisons of normal distributed variables were performed using one-way analysis of variance (ANOVA). Those results found to be significant were compared with Post-hoc analysis Tukey HSD test. Comparison of data with non-normal distribution was performed with Kruskal-Wallis test. Categorical variables were evaluated with chi-square analysis. A *p* value of less than 0.05 was accepted as statistically significant.

## RESULTS

There were no statistically significant difference between groups regarding the demographic data (age, weight, gender), number of extracted teeth and operation times (p>0.05) (Table I and II). Recovery time was significantly lower in Group P (8.2±4.6 min) than Group KP (15.5±7.1 minutes) (p=0.001) (Table-I). Surgeon satisfaction was significantly higher in Group KP compared to the others (p= 0.003) (Table-II).

**Table-I:** The demographic data, operation time, recovery time of the groups.

	*Group K (n=20) (X±SD)*	*Group P (n=20) (X±SD)*	*Group KP (n=20) (X±SD)*	*P*
*Age (year)*	5.6±1.6	4.9±2.0	6.3±1.7	0.07
*Gender (M/F)*	10 /10	8 /12	7 / 13	0.61
*Weight (kg)*	21.2±6.1	20.1±7.1	23.6±7.3	0.25
*Operation time (min)*	6.5±2.3	5.9±1.5	6.6±2.3	0.502
*Recovery Time (min)*	11.2±5.1	8.2±4.6*	15.5±7.1	0.001

* significant decrease compared with Group KP.

**Table-II:** Surgeon satisfaction, number of extracted teeth, numbers of anesthetic dose repetitions, postoperative anxiety scores.

	*Group K (n=20)*	*Group P (n=20)*	*Group KP (n=20)*	*P*
Surgeon satisfaction (bad/middle/good)	5/6/9	0/9/11	0/3/17*	0.003
Number of extracted teeth (1/2/>2)	4/6/10	6/7/7	2/6/12	0.678
Numbers of anesthetic dose repetitions (-/+)	7/13**	14/6	16/4	0.009
Postoperative anxiety scores: calm/anxiosus/could not be reassured/crying	17/2/1/0	16/3/0/1	18/12/0/0†	0.006

* Better than the others,

** Significantly higher than Group KP,

† Postoperative anxiety scores worse in Group KP.

**Fig.1:**
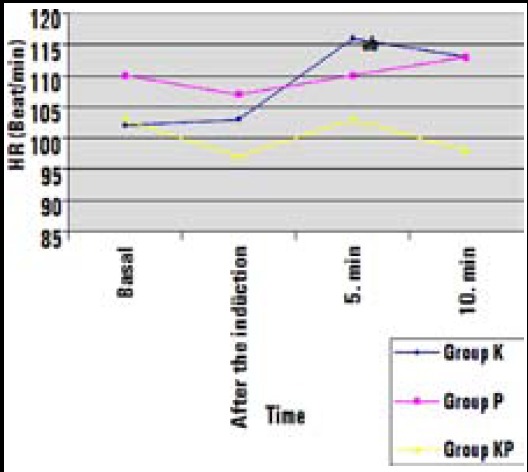
Heart Rate of the groups (beat/min) ^a^Higher in Group K than Group KP, ^b^Significant increase compared with baseline.

**Fig.2:**
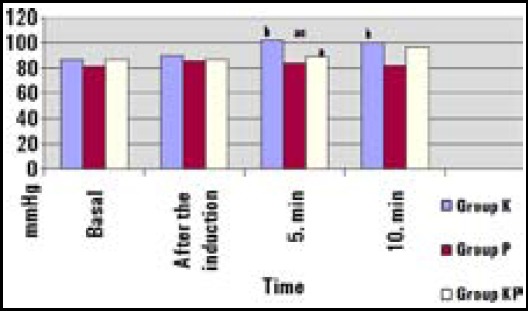
Mean arterial pressure of the groups (mmHg). ^a^Significant decrease compared with Group K, ^c^Significant decrease compared with Group KP, ^b^Significant increase compared with basaline

No significant differences were found between groups regarding HR, before and after the induction, and at tenth minute (p>0.05). However, at fifth minute, HR was significantly higher in Group K than in Group KP (p=0.020). In Group K, at fifth and tenth minutes, HR were higher than baseline values (p=0.001 and p=0.038) (Fig.1). Mean arterial pressure (MAP) values were similar at baseline, before and after the induction, and at tenth minute, whereas significantly lower values were found in Group P and Group KP than in Group K at fifth minute (p<0.001). In Group K, MAP values were increased when compared to baseline values at fifth and tenth minutes (p=0.007 and p=0.019) (Fig.2).

There were no statistically significant differences between the groups regarding respiratory rate, and end-tidal CO_2_ values at all times (p>0.05). There were no statistically significant difference of SPO_2_ at any time (p>0.05). There were no statistically significant difference of Ramsey Sedation Scores at any time (p>0.05). Postoperative anxiety score was worsen in Group KP due to higher anxious patients compared to Groups K and P(p=0.006) (Table-II). Additional drug repetitions was not different between Group K and P, however it was significantly higher in Group K than in Group KP (p= 0.057 and p=0.009) (Table-II). Postoperative nausea and vomiting was seen in seven patients in Group K, four patients in Group KP (p=0.016) and none in Group P. Respiratory depression was observed only in Group P in three patients (p= 0.043), but not in the others. Tachycardia was observed in one patient in each group (p>0.05).

## DISCUSSION

In this study, we concluded that ketamine, propofol and ketamine-propofol combination were effective for intravenous deep sedation in tooth extraction, in non-cooperative children with severe anxiety. Although ketamine-propofol combination provided better surgical satisfaction levels, it required longer recovery times. Moreover, propofol may be an excellent alternative, with the shortest recovery, no nausea and vomiting, and reasonable surgical satisfaction.

Intravenous sedation reduces dental anxiety, provides a comfortable and reliable surgical environment.^12^ Most of the dental procedures can be performed with non-pharmacological behavioral therapies in children in dental clinics.^2^ When these methods do not work, children often are treated with conscious sedation through administration of nitrous oxide inhalation. Nitrous oxide inhalation provides successful results in children with milder anxiety levels, however, the success rate is low with severe anxiety, leading to repeated procedures.^3^,^12^ For patients with severe anxiety, deep sedation and general anesthesia may be necessary for dental treatments. Considering the low efficiency, and potential side effects of nitrous oxide, deep sedation with intravenous agents may be a better option for dental treatments such as tooth extraction in pediatric patients.

Ketamine and propofol, either alone or in combination, have been successfully used for pediatric patients in various invasive procedures for the years.^5^ Hosey et al.^14^ reported that sub-anesthetic doses of propofol are effective in the dental treatments of anxious children. Wood et al.^15^ successfully used midazolam and ketamine in dental treatments of 500 children. In this recent study, we used propofol, ketamine and the combination of them to facilitate dental treatment in children by achieving deep sedation.

Guit et al.^16^ determined that propofol eliminates the side effects of ketamine at sub-anesthetic doses, and ketamine-propofol combination provides hemodynamic stability. In our present study, it has been observed that the heart rate was higher in the ketamine group at fifth minute when compared to that of KP group. The fact that the heart rate and mean arterial pressure values increased at fifth and tenth minutes when compared to basal line values, and higher MAP values were determined at fifth minute in ketamine group than in other groups. This result may be because ofsympathomimetic effect of ketamine. In this study, hemodynamic stability was better in KP group than only ketamine or propofol groups.

Lebovicet al.^17^ concluded that the recovery time of pediatric patients with cardiac catheterization is shorter when propofol is used, when compared to ketamine, and suggested it for daily operations as a more practical alternative. In a clinical study, Akin et al.^18^ compared propofol and ketamine-propofol combination in cardiac catheterization operation for pediatric patients, and found that ketamine-propofol combination provided better mean arterial pressure without affecting the recovery. Accordingly, when the effects of ketamine, propofol and ketofol for electroconvulsive treatment were compared in adult patients, ketofol provided better hemodynamic stability, whereas ketamine leaded to longer recovery time.^19^ In the present study, at fifth and tenth minutes HR and MAP values in ketamine group were higher than baseline values. At fifth minute, MAP was lower in propofol and ketamine-propofol groups than ketamine group. This might be due to sympathomimetic effect of ketamine. Although these hemodynamic changes were statistically significantly, they appeared to be insignificant.

Kramer et al. concluded that there is no difference in surgical satisfaction and patient satisfaction levels in propofol-remifentanyl and propofol-ketamine groups for third molar tooth treatment under deep sedation. However, they suggested propofol-remifentanyl to be a better option in ambulatory procedures for shorter recovery times.^21^ In our study, however, ketamine-propofol and only propofol provided a more convenient operation resulting in a better surgical satisfaction. Therefore, surgeon satisfaction was worsening with only ketamine.

Respiratory depression and hypoxia may occur when propofol is not well titrated. In addition, the incidence of airway complications decreased when it was used in conjunction with ketamine.^5^ Erden et al. detected that low dose ketamine addition to fentanyl-propofol combination decreases desaturation risk, and additional propofol requirement during radiological procedures.^22^ In the present study, no difference was found between the groups regarding SPO_2_ respiration rate and end-tidal CO_2_ values. In a controlled study for procedural sedation for management of orthopedic extremity injury, lower levels of nausea were detected when propofol-ketamine combination was used compared to ketamine alone.^25^ Likewise, in our study, postoperative nausea and vomiting were observed in 7 patients in ketamine group, in 4 patients in ketamine-propofol group, and none in propofol group. This may be due to the antiemetic feature of propofol.

In conclusion, although ketamine, propofol and ketamine-propofol combination are effective for sedation in tooth extraction in pediatric patients, propofol may be an excellent alternative, with the shortest recovery, no nausea and vomiting, and reasonable surgeon satisfaction.
